# Biomarkers of extracellular matrix turnover are associated with emphysema and eosinophilic-bronchitis in COPD

**DOI:** 10.1186/s12931-017-0509-x

**Published:** 2017-01-19

**Authors:** Asger Reinstrup Bihlet, Morten Asser Karsdal, Jannie Marie Bülow Sand, Diana Julie Leeming, Mustimbo Roberts, Wendy White, Russell Bowler

**Affiliations:** 1 Nordic Bioscience A/S, Herlev Hovedgade 207, DK-2730 Herlev, Denmark; 2 Bristol-Meyers Squibb, 3551 Lawrenceville, Lawrence Township, NJ 08648 USA; 3 MedImmune, LLC, One MedImmune Way, Gaithersburg, MD 20878 USA; 4 National Jewish Health, Denver, Colorado, 1400 Jackson Street, Room K715a, Denver, CO 80206 USA

**Keywords:** COPD, Emphysema, Extracellular matrix, Biomarkers, Eosinophils

## Abstract

**Background:**

Chronic obstructive pulmonary disease (COPD) is characterized by airflow obstruction and loss of lung tissue mainly consisting of extracellular matrix (ECM). Three of the main ECM components are type I collagen, the main constituent in the interstitial matrix, type VI collagen, and elastin, the signature protein of the lungs. During pathological remodeling driven by inflammatory cells and proteases, fragments of these proteins are released into the bloodstream, where they may serve as biomarkers for disease phenotypes. The aim of this study was to investigate the lung ECM remodeling in healthy controls and COPD patients in the COPDGene study.

**Methods:**

The COPDGene study recruited 10,300 COPD patients in 21 centers. A subset of 89 patients from one site (National Jewish Health), including 52 COPD patients, 12 never-smoker controls and 25 smokers without COPD controls, were studied for serum ECM biomarkers reflecting inflammation-driven type I and VI collagen breakdown (C1M and C6M, respectively), type VI collagen formation (Pro-C6), as well as elastin breakdown mediated by neutrophil elastase (EL-NE). Correlation of biomarkers with lung function, the SF-36 quality of life questionnaire, and other clinical characteristics was also performed.

**Results:**

The circulating concentrations of biomarkers C6M, Pro-C6, and EL-NE were significantly elevated in COPD patients compared to never-smoking control patients (all *p* < 0.05). EL-NE was significantly elevated in emphysema patients compared to smoking controls (*p* < 0.05) and never-smoking controls (*p* < 0.005), by more than 250%. C1M was inversely associated with forced expiratory volume in 1 s (FEV_1_) (*r* = −0.344, *p* = 0.001), as was EL-NE (*r* = −0.302, *p* = 0.004) and Pro-C6 (*r* = −0.259, *p* = 0.015). In the patients with COPD, Pro-C6 was correlated with percent predicted Forced Vital Capacity (FVC) (*r* = 0.281, *p* = 0.046) and quality of life using SF-36. C6M and Pro-C6, were positively correlated with blood eosinophil numbers in COPD patients (*r* = 0.382, *p* = 0.006 and *r* = 0.351, *p* = 0.012, respectively).

**Conclusions:**

These data suggest that type VI collagen turnover and elastin degradation by neutrophil elastase are associated with COPD-induced inflammation (eosinophil-bronchitis) and emphysema. Serological assessment of type VI collagen and elastin turnover may assist in identification of phenotypes likely to be associated with progression and amenable to precision medicine for clinical trials.

## Background

The current treatment options for disease modification in COPD are limited [1, 2]. Lack of progress in drug development may be due to a lack of identification of the optimal patient for the optimal intervention (i.e., precision medicine) [3]. However, the recent approvals of roflumilast for subsets of COPD patients demonstrate a feasible precision medicine approach in which patients with severe or very severe COPD associated with chronic bronchitis and a history of exacerbations showed a statistically significant reduction in exacerbations, when given as an add-on to combined inhaled therapies [1, 4, 5].

Another factor limiting clinical trial feasibility is the slow progression of COPD, exemplified by the modest declines observed in pre-bronchodilator Forced Expiratory Volume in 1 s (FEV_1_) of the trials comparing roflumilast to placebo [6]. A similarly small decrease was observed also in the Evaluation of COPD Longitudinally to Identify Predictive Surrogate Endpoints (ECLIPSE) observational study. From this study, Vestbo and colleagues reported an average annual FEV_1_ decline of 33 mL/year, and further found that a notable proportion of patients did not experience a decline in lung function [7]. Data derived from multiple large clinical trials indicate that the average lung function decline appears to be higher in an earlier stage (GOLD 2) of COPD, compared to later stages (GOLD 3 and 4) [8–12]. In direct alignment with this, a number of COPD patients may never have undergone a significant loss of lung function in terms of FEV_1_, but may have had a low lung function in early adulthood, raising the risk of ultimately having significant airflow limitation despite a normal or only slightly decline in FEV_1_ over time [13–16].

Despite significant investments made in identifying genetic factors which may influence either COPD disease development or severity, only a minor proportion of patients carry identifiable genetic anomalies such as severe alpha-1 antitrypsin (AAT) deficiency, which has been shown to significantly influence development of emphysema [15]. Large-scale attempts to identify biomarkers reflecting COPD subtypes have yielded modest results [17, 18], in which cytokines and air pollution may provide some value as predictive markers for progression [19, 20], albeit new biomarkers are needed [3].

Recently, an increased attention to identification of phenotypes in COPD has been pursued consequent to the lack of success in drug development in broader disease populations with functional modulators [3]. Several researchers have suggested changing the respiratory phenotypes into more targetable and treatable traits [21]. Two potential phenotypes include: emphysema and the eosinophil-bronchitis, however for their identification and monitoring simple serological biomarkers are lacking. Currently, the phenotyping is mainly based on a combination of clinical and morphological features such as type and severity of symptoms [22, 23]. Results from the ECLIPSE study suggest that a frequent-exacerbator phenotype exists, irrespective of disease severity, and that the best predictor of future exacerbations is a history of exacerbations [24], albeit others did not replicate this finding and found smoking to be the only predictor of acute respiratory episodes [25]. Further complicating patient reported outcomes, published reports indicate significant variability of these measures, perhaps due to failure of the patient to identify exacerbations caused by diffuse symptomatology or lack of clear association between symptoms and event from a patient perspective, ultimately leading to underreporting [24–26]. Imaging assessments of the lung parenchyma and airways using computed tomography (CT) are only feasible in a subgroup of the population preselected for having COPD diagnosed by spirometry and symptoms [27], limiting the potential for screening of phenotype identification.

Chronic inflammation in the lungs leads to repeated cycles of injury and repair of the airway walls [28–30]. Elevated concentrations of inflammatory markers in blood are also able to predict groups with a higher risk of future exacerbations [31], but none of these modalities have been approved for standard clinical care for individual patients. Possibly a new form of inflammatory and structural biomarkers may provide value, such as biomarkers of tissue turnover driven by inflammation [32–34]. A central part of lung function decline is extracellular matrix (ECM) remodeling [35]. During structural remodeling of the airway walls, an increase in ECM protein deposition and scar tissue formation results in narrowing of the lumen and airway obstruction, resulting in functional loss [36]. ECM turnover is a delicate balance between formation and degradation. It is considered an important element in tissue homeostasis, in which old proteins are continuously degraded and new proteins are formed [37]. This equilibrium is out of balance in diseases affecting connective tissue, and in the case of COPD, may results in an increase in both formation and degradation of tissue in the peripheral airway wall which may lead to tissue disruption and fibrosis [32, 38].

Previous research shows that the airway wall composition is changed in patients suffering from COPD as compared to healthy individuals, in which an increased deposition of type I and III collagens, fibronectin, and laminin have been identified [39, 40], along with the proteoglycans, perlecan decorin, versican and biglycan [41]. Some proteases have been reported to be over-expressed in tissue affected by COPD, such as elastase [42] and matrix metalloproteinases (MMP)-1, -2, -7 and -12 [43], of which most are collagenolytic. Their activity results in the release of protease-specific fragments of ECM proteins. It is recognized that in emphysema, both elastin and collagen degradation in alveoli occurs [40], thus generating elastin- and collagen fragments which are released into the systemic circulation. These protease-derived protein fragments may be used as serological biomarkers of tissue formation or degradation, reflecting the type of remodeling activity [33, 44, 45], and therefore have the potential to be used as diagnostic or prognostic tools if adequately validated. Examples of biomarkers of remodeling of structural proteins are MMP-2, -9, and 13-mediated destruction of interstitial type I, III, V, and VI collagen [46–48] and the basement membrane type IV collagen [49], which have all been found to be associated with connective tissue diseases.

The aim of this study was to investigate the degree of lung ECM remodeling in healthy smokers and non-smokers and COPD patients from a sub-group of the COPDGene study, possibly associated with the two major phenotypes in respiratory diseases, the emphysema and eosinophil-predominant (Bronchitis) phenotypes. We focused on the signature protein of the lung, elastin, degraded by neutrophil elastase [50], degradation of the main component of lung interstitial matrix, type I collagen, and remodeling of type VI collagen, found at the interface of the basement membrane and interstitial matrix [37], which is disrupted during progression of COPD [35].

## Methods

### Study population

This study was approved by the Independent Review Board and all patients gave informed written consent. The basis of this analysis is a cross-sectional post-hoc investigation of a subset of the COPDGene study. The COPDGene study recruited 10,300 COPD patients in 21 centers (see [51]). The major inclusion criteria were non-Hispanic white or African-American race, age between 45–80 years and at least 10 years of smoking history. Major exclusion criteria include a history of other non-asthma lung diseases, lung cancer, surgical resection of one or more lung lobe, or COPD exacerbation within 1 month prior to inclusion. All patients underwent a clinical examination including blood sampling, spirometry to assess lung function, questionnaires to assess the quality of life (The Short Form 36 (SF-36) [52]), and CT-scan upon inclusion in the trial. In one center, a subset of 89 subjects including 52 COPD patients, 12 never-smoker controls and 25 smokers without COPD by spirometry (FEV_1_/FVC ratio > 0.70) were asked to participate and provide additional blood for studies of ECM.

Clinical definitions were as follows: Emphysema was defined as a low attenuation area at −950 Hounsfield Units (%LAA) >5% on chest CT scans, and chronic bronchitis defined as having current symptoms of chronic bronchitis in addition to COPD by spirometry. The Chronic Bronchitis-phenotype was defined in accordance with the definition by GOLD as the presence of cough and sputum production for at least 3 months in each of two consecutive years [53] in addition to COPD by spirometry. Never-smokers were defined as patients having smoked less than 100 cigarettes in their lifetime. Smoker controls had to have a smoking history of at least 10 pack years.

ECM-related biomarkers of type I collagen degradation by MMPs (C1M), type VI collagen degradation by MMPs or formation (C6M, Pro-C6) and elastin degraded by neutrophil elastase (EL-NE) were measured in serum samples from the 89 subjects using highly specific Enzyme-Linked Immunosorbent Assays (ELISAs) for such fragments. Monoclonal antibodies against specific protein fragments resulting from proteolytic cleavage by a specific protease were used in each ELISA; a description of each assay is listed in Table 1.

**Table 1:** Overview of biomarkers measured, description and biological relevance

	Biomarker description	Biological relevance	References
C1M	Fragment of type I collagen degraded by MMPs	Inflammatory interstitial matrix destruction	[46]
C6M	Fragment of type VI collagen degraded by MMPs	Inflammatory interstitial matrix destruction	[70]
Pro-C6	Pro-peptide of type VI collagen	Formation of new interstitial matrix	[66]
EL-NE	Fragment of elastin degraded by neutrophil elastase	Inflammatory destruction of interstitial matrix	[50]

*MMP* matrix metalloproteinase

### Statistical analysis

The mean concentrations of the respective biomarkers was compared between COPD (*n* = 52) and control patients smoking (*n* = 25) and never-smoking (*n* = 12) as well as three selected subgroups of the study population; 1: Patients with diagnosed emphysema (*n* = 30), 2: Patients with both chronic bronchitis and emphysema (mixed disease, *n* = 15), and 3: an “obstructive” phenotype with the absence of emphysema and chronic bronchitis, yet COPD as assessed by spirometry, defined as FEV_1_/FVC < 0.70 (*n* = 7). Six COPD patients, and 6 smoking controls were current smokers at the time of this analysis. Mean values between subgroups were compared using one-way ANOVA, and multiple comparisons by Tukey’s multiple comparison test, with an alpha of 0.05. Correlation of biomarkers with lung function, the SF-36 quality of life patient reported outcome, and haematology test results including eosinophil counts were performed using Spearman’s correlation.

## Results

### Demographics

The mean age of COPD patients was 69.5 years (inter-quartile range (IQR): 66–75), while the control patients had a mean age of 63.6 years (IQR 56–70). Fifty-two and 24% of COPD patients and controls were male, respectively. An overview of important demographic and clinical characteristics is shown in Tables 2 and 3.

**Table 2:** Main clinical characteristics of the study population

	COPD *n* = 52	Controls *n* = 37	Total *n* = 89
Mean age, years (IQR)	69.5 (66–75)	63.6 (56–70)	66.7 (60.3–74)
Male sex, *n* (%)	27 (52)	9 (24.3)	35 (39.3)
Current smokers, *n* (%)	5 (9.6)	12 (32.4)	17 (19.1)
BMI (kg/m^2^)	29.3 (9.4)	28.6 (9.6)	29.0 (9.4)
GOLD stage, *n* (%)			
1	7 (13.5)	N/A	N/A
2	16 (30.7)	N/A	N/A
3	12 (23.1)	N/A	N/A
4	16 (30.7)	N/A	N/A
N/A	1 (1.9)	N/A	N/A
FEV_1_, liters (SD)	1.47 (0.77)	2.60 (0.77)	1.95 (0.95)
FEV_1_, % of predicted (SD)	58.4 (74.4)	92.5 (16.4)	83.1 (47.6)
FVC, liters (SD)	2.71 (1.06)	3.25 (0.90)	2.94 (1.02)
FVC, % of predicted (SD)	77.5 (60.0)	89.9 (19.2)	83.1 (48.6)
FEV_1_/FVC ratio	0.49 (0.13)	0.80 (0.05)	0.62 (0.19)
6 MWD, meters (SD)	340 (134)	455 (110)	391 (137)
SF36 PCS (SD)	36.3 (10.6)	50.8 (8.2)	42.4 (12.0)
SF36 MCS (SD)	53.4 (9.1)	52.6 (11.4)	53.1 (10.1)

Data are presented as mean (SD), unless stated otherwise. *IQR* Inter-Quartile Range, *BMI* Body Mass Index, *FEV*_*1*_ Forced Expiratory Volume in 1 s, *FVC* Forced Vital Capacity, *6MWD* 6 min Walking Distance, *SF 36 PCS* Physical Component Score of the SF-36 quality of life psychometric tool, *SF-36 MCS* Mental Component Score of the SF-36 quality of life psychometric tool. Higher score reflects better health

**Table 3:** Main clinical characteristics of the study population, by phenotype/subgroup

Phenotype/subgroup	Chronic bronchitis/emphysema (*n* = 15)	Emphysema (*n* = 30)	Obstructive (*n* = 7)	Smoking control (*n* = 25)	Never-smoking control (*n* = 12)	Total *n* = 89
Mean age, years (IQR)	66.3 (59.5–72.5)	70.5 (67–76.5	71.4 (67–72.5)	64.2 (56–71)	62.3 (54–69.3)	66.7 (60.3–74)
Male sex, *n* (%)	7 (46.7)	16 (53.3)	4 (57.1)	5 (20)	4 (33.3)	35 (39.3)
Current smokers, *n* (%)	3 (20)	1 (3.3)	1 (14.3)	12 (48)	0 (0)	17 (19.1)
BMI (kg/m^2^)	30.1 (8.5)	29.5 (10.5)	27.1 (4.2)	30.4 (10.9)	24.8 (3.6)	29.0 (9.4)
GOLD stage *n*, %)				N/A		
1	3 (20.0)	2 (6.7)	2 (28.5)			7 (7.9)
2	2 (13.3)	11 (36.7)	3 (42.9)			16 (18.0)
3	3 (20.0)	7 (23.3)	2 (28.6)			12 (13.5)
4	7 (46.7)	9 (30.0)	0 (0)			16 (18.0)
N/A	0 (0)	1 (3.3)	0 (0)			1 (1.1)
FEV_1_, liters (SD)	1.29 (0.80)	1.39 (0.61)	2.16 (0.88)	2.51 (0.73)	2.80 (0.80)	1.95 (0.95)
FEV_1_, % of predicted (SD)	46.9 (29.9)	62.8 (95.4)	65.1 (17.2)	90.2 (17.5)	97.2 (12.4)	83.1 (47.6)
FVC, liters (SD)	2.77 (1.00)	2.54 (0.98)	3.32 (1.27)	3.17 (0.90)	3.42 (0.86)	2.94 (1.02)
FVC, % of predicted (SD)	74.9 (37.2)	79.7 (74.4)	73.9 (18.6)	92.0 (12.4)	85.7 (28.0)	83.1 (48.6)
FEV_1_/FVC ratio	0.43 (0.14)	0.49 (0.12)	0.65 (0.02)	0.79 (0.06)	0.81 (0.04)	0.62 (0.19)
6 MWD, meters (SD)	298 (162)	317 (162)	415 (139)	421 (104)	527 (85)	391 (137)
SF36 PCS	33.3 (10.2)	36.7 (10.0)	41.0 (11.5)	49.3 (8.9)	54.0 (5.2)	42.4 (12.0)
SF36 MCS	47.6 (9.9)	55.0 (8.1)	58.9 (3.0)	50.9 (12.8)	56.0 (6.6)	53.1 (10.1)

Data are presented as mean (SD), unless stated otherwise. *IQR* Inter-Quartile Range, *BMI* Body Mass Index, *FEV*_*1*_ Forced Expiratory Volume in 1 s, *FVC* Forced Vital Capacity, *6MWD* 6 min Walking Distance, *SF-36 PCS* Physical Component Score of the SF 36 quality of life psychometric tool, *SF 36 MCS* Mental Component Score of the SF 36 quality of life psychometric tool. Higher score reflects better health

### Biomarkers associated with COPD

**Fig. 1:**
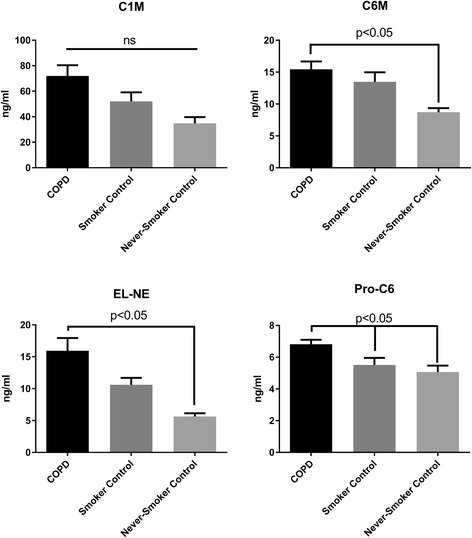
Mean biomarker concentrations of patients with chronic obstructive pulmonary disease and controls. One-way ANOVA test of differences between patients and controls were made using Tukey’s multiple comparisons test. C1M: Type I collagen degraded by matrix metalloproteinases. C6M: Type VI collagen degraded by matrix metalloproteinases. Pro-C6: Pro-peptide fragment of type VI collagen. EL-NE: Elastin degraded by neutrophil elastase

The circulating concentrations of biomarkers reflecting type VI collagen turnover (C6M and Pro-C6) and elastin degradation by neutrophil elastase (EL-NE) were significantly elevated in COPD patients compared to never-smoking controls (all *p* < 0.05) (Fig. 1). C6M was also significantly elevated compared to smoking controls (*p* < 0.05). No significant differences in circulating biomarker concentrations were identified between smoking- and never-smoking controls. Differences in C1M between COPD patients and smoking or non-smoking controls were statistically significant using a standard one-way ANOVA (*p* = 0.044), but not in multiple comparison testing using Tukey’s (Fig. 1). The mean concentration of C1M was 71.99 ng/ml in COPD patients compared to 34.78 ng/ml in never-smokers (95% CI of difference: −1.285 to 75.72 ng/ml).

### Biomarkers associated with COPD subgroups

**Fig. 2:**
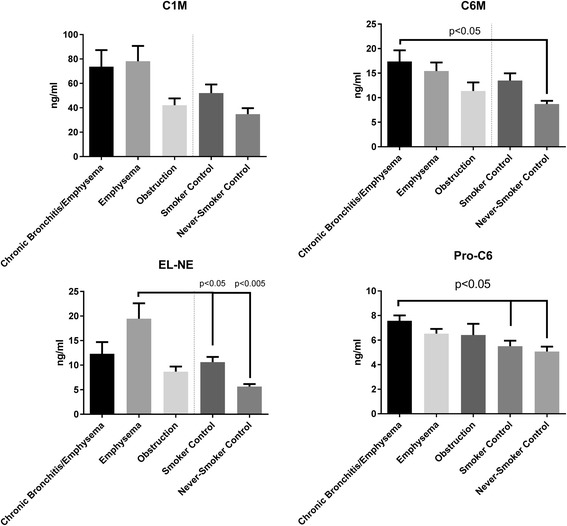
Mean biomarker concentrations per sub-group. One-way ANOVA test of differences between COPD phenotypes and controls were made using Tukey’s multiple comparisons test. The Mixed disease group (“Chronic Bronchitis/Emphysema”, *n*=15) was defined as emphysema as diagnosed using CT and chronic bronchitis. The Emphysema group was defined as diagnosed emphysema using CT in the absence of chronic bronchitis (*n* = 30). The obstruction group was defined as the absence of both emphysema and chronic bronchitis, yet with the presence of significant respiratory obstruction diagnosed using spirometry (*n* = 7). C1M: Type I collagen degraded by matrix metalloproteinases. C6M: Type VI collagen degraded by matrix metalloproteinases. Pro-C6: Pro-peptide fragment of type VI collagen. EL-NE: Elastin degraded by neutrophil elastase

In the group of patients with mixed disease, defined as having both chronic bronchitis and emphysema, C6M and Pro-C6 were both found to be statistically significantly elevated (*p* < 0.05) compared to never-smoking controls (Fig. 2). Pro-C6 was also significantly elevated among patients with mixed disease compared to smoking controls (*p* < 0.05).

EL-NE was particularly associated with emphysema only, as the concentrations of this biomarker was significantly elevated in this subgroup compared to smoking controls (*p* < 0.05) and never-smoking controls (*p* < 0.005), but was not found to be significantly elevated in mixed disease and obstructive, non-emphysematous, COPD compared to controls (Fig. 2).

C1M was not statistically significantly elevated in either of the groups, but a trend towards elevated C1M concentrations in patients with chronic bronchitis and/or emphysema was noted, whereas the concentration of C1M in the group of obstructive patients, non-emphysematous patients without chronic bronchitis, was similar to that of smoking and never-smoker controls (Fig. 2).

### Associations between biomarkers, blood cell counts, and other clinical characteristics

C6M and Pro-C6 were positively correlated with blood eosinophil numbers in COPD patients (*r* = 0.382, *p* = 0.006 and *r* = 0.351, *p* = 0.012, respectively). In the total study of COPD patients and controls, C1M was inversely associated with % of predicted FEV_1_ (*r* = −0.344, *p* = 0.001), as was EL-NE (*r* = −0.302, *p* = 0.004) and Pro-C6 (*r* = −0.259, *p* = 0.015). Notably, these correlations were not found to be significant in the group of COPD only (Table 4). In the patients with COPD, Pro-C6 was significantly positively correlated to % of predicted FVC (*r* = 0.281, *p* = 0.046). C6M was inversely correlated to 6-min walking distance (*r* = −0.311, *p* = 0.028) in COPD patients. In COPD patients, Pro-C6 was also inversely correlated to the SF-36 quality of life Physical Component Score (PCS) (−0.361, *p* = 0.009) and Mental Component Score (MCS) (−0.308, *p* = 0.028).

**Table 4:** Spearman’s correlations between biomarkers, clinical characteristics and blood cell counts in COPD patients regardless of subtype (*n* = 52)

	FEV_1_ % of predicted	FVC % of predicted	6MWD	SF36 PCS	SF36 MCS	Eosinophils
C1M						
*r*	−0.252	−0.253	−0.271	−0.187	0.037	0.085
*p*-value	0.08	0.07	0.05	0.19	0.80	0.55
EL-NE						
*r*	−0.060	−0.163	−0.274	−0.125	0.137	0.204
*p*-value	0.65	0.25	0.05	0.09	0.33	0.15
C6M						
*r*	−0.091	−0.149	**−0.311**	−0.242	0.130	**0.382**
*p*-value	0.53	0.30	**0.028**	0.09	0.37	**0.006**
Pro-C6						
*r*	0.239	**0.281**	−0.207	**−0.361**	**−0.308**	**0.351**
*p*-value	0.09	**0.046**	0.14	**0.009**	**0.028**	**0.012**

Correlations with a *p*-value ≤ 0.05 are highlighted in bold. *FEV*_*1*_ Forced Expiratory Volume in 1 s, *FVC* Forced Vital Capacity, *6MWD* 6 min Walking Distance, *SF 36 PCS* Physical Component Score of the SF 36 quality of life psychometric tool, *SF-36 MCS* Mental Component Score of the SF-36 quality of life psychometric tool. Higher score in the SF-36 reflects better health

## Discussion

This study identifies associations between specific protein fragments of lung ECM constituents and major clinical manifestations of COPD. The association between fragments of elastin, as degraded by neutrophil elastase, and emphysema is particularly interesting as they point to potential targets of pathological tissue remodeling in certain phenotypes of COPD which have not been described before. Moreover, the associations between type VI collagen turnover and eosinophils, eosinophil-bronchitis, suggest a biological interaction between matrix turnover and inflammation, localized in the interface between the basement membrane and interstitial membrane, a well-known site for chronic inflammation.

Elastin is a structural protein abundant in lung tissue where it provides resilience and elasticity to the lungs [54], and is of particular interest to the emphysema phenotype, as this is the main protein with predominant expression in the lung, a so-called signature protein, which is degraded during lung inflammation and destruction [35, 50, 55, 56]. Neutrophils have received increased attention for their role in chronic inflammation and wound healing, in addition to their role in the primary inflammatory response [50]. When neutrophils degrade the surrounding matrix by neutrophil elastase a specific fragment of elastin, EL-NE, is generated and released into the circulation [50]. As a part of the technical validation of the biomarker for the use of quantification of EL-NE, the antibody raised to capture this fragment was found in vitro to be capable of binding only the EL-NE fragment, with no binding of intact elastin, nor fragments of elastin degraded by MMP or cathepsin G [50] Consequently, this biomarker may be associated with the emphysema phenotype.

**Fig. 3:**
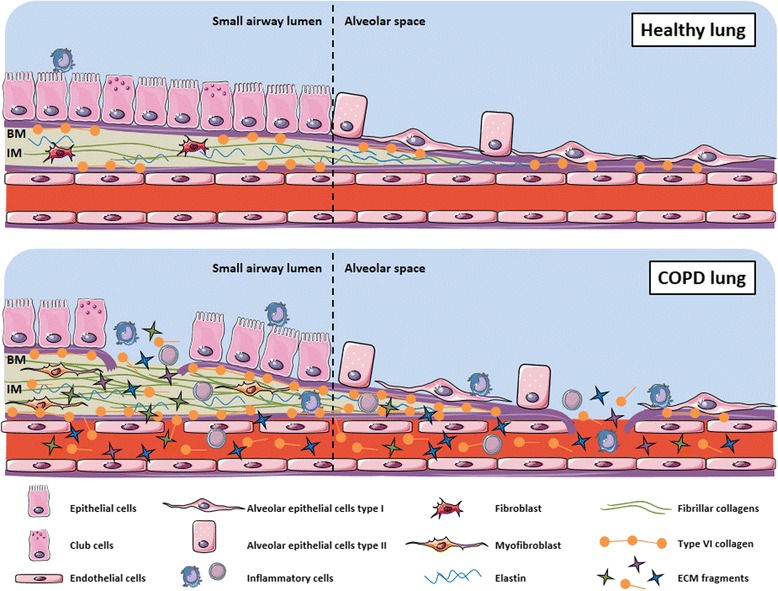
Remodeling of the lung extracellular matrix. During progression of COPD, the basement membrane (BM) and the interstitial matrix (IM) is remodeled, resulting in a disruption of the interface between these two extracellular matrix (ECM) compartments. Type VI collagen is situated in this interface, and consequently fragments of type VI collagen, may be particularly related to disease progression and lung tissue remodeling. In addition, the chronic inflammatory response may in part be accompanied by persistent neutrophil presence in affected tissues, which normally would be resolved in the later stages of inflammation resolution. These inflammatory cells produce high amounts of neutrophil elastase capable of degrading the elastin of the lungs found in the interstitial matrix, which is essential for lung tissue elasticity. Both the type VI collagen and elastin remodeling, in addition to the remodeling of other ECM components, results in the release of small protein fragments (neo-epitopes) to the bloodstream. Modified with permission from [65]

Smoking induces elevated levels of neutrophils and macrophages in the lung [57]. During acute lung injury, the neutrophils produce the serine protease elastase which is able to degrade the majority of ECM proteins including the otherwise stable elastin fibres [58, 59], resulting in the biomarker EL-NE [50]. It is possible that smoking may increase the concentrations of elastases and collagenases and decrease the concentrations of anti-proteinases such as AAT. An important notion of elastin research is that AAT is the main inhibitor of neutrophil elastase, and AAT deficiency leads to the development of emphysema in smokers at a relatively young age [60, 61]. The protease-antiprotease imbalance in emphysema leads to unopposed elastolysis by neutrophil elastase [60]. This has been confirmed by comparing CT investigations of emphysema with assessments of elastase and anti-elastase activity in bronchoalveolar lavage fluid from COPD patients [62]. The study demonstrated that the activity of neutrophil elastase correlated to emphysema whereas the AAT activity correlated inversely with emphysema [62]. Previous studies have found a significantly elevated concentration of elastin degradation-specific amino acids, desmosine and isodesmosine, in asymptomatic individuals with known exposure to second-hand smoke and smokers as compared to non-smokers [63]. Further, highly increased expression of elastin has been found in the alveoli of severe COPD patients [64]. The results of this analysis support the findings of previous reports, as serological concentrations of fragments of collagen and elastin were higher in patients with various clinical manifestations of COPD, and particularly the association between EL-NE is promising, as it could indicate that this biomarker has a potential to aid in a non-invasive, inexpensive method of emphysema diagnosis. This hypothesis will need to be validated in further studies. In addition to elastin, other ECM proteins are important for upholding lung structure and function. These maintain the interface between the basement membrane and interstitial matrix of the airways, the epithelium and endothelium interrelationship, and in particular the interstitial matrix [35]. Figure 3 illustrates the ECM remodelling occurring in COPD lungs and the resulting release of small protein fragments (neo-epitopes) into the systemic circulation. The main protein of the interstitial matrix is the fibrillar type I collagen, which during inflammation is degraded by MMPs, in part originating from macrophages and other inflammatory cells, resulting in the fragment C1M [46]. This fragment is released into the systemic circulation and may be used as a biomarker of interstitial matrix destruction. While this report did not find significantly elevated concentrations of C1M in COPD, one previous reports did [44], and a recent report found significant associations of C1M with risk of mortality in COPD patients [65]. Based on the current results it is likely that, using a larger sample size, a similar association between C1M and COPD could have been found.

Type VI collagen, a protein very different from the fibrillar collagen types, is interconnecting proteins and membranes. Type VI collagen is a unique beaded filament collagen, with a special structure forming micro-filament networks, found in the interface between the basement membrane and the interstitial matrix [46]. Type VI collagen has many binding partners and is part of the backbone of the matrix [66]. In addition to these important roles, the pro-peptide of type VI collagen is now also recognized as a hormone involved in metabolic dysfunction, named endotrophin [67–69]. Degradation by MMPs results in the release of C6M [70], and formation of the same molecule results in release of Pro-C6 [66]. The results of the correlations of C6M and Pro-C6 with COPD indicate that high degradation and formation of type VI collagen may in part be a biochemical reflection of important clinical features of COPD, including poor ability to walk, as assessed by the 6-min walk test (C6M) and generally poorer quality of life reflected in lower scores of physical and mental well-being (Pro-C6). Other reports have previously found associations of collagen degradation with COPD. Proline-glycine-proline (PGP), a tripeptide neutrophil chemoattractant originating from collagen degradation, was found to be elevated in sputum of patients with COPD exacerbations, and reduced by azithromycin treatment [71]. Of special importance with respect to the current findings, is that both type VI collagen formation (Pro-C6) and degradation (C6M) were correlated to eosinophil blood count. This is an interesting finding as in both asthma and COPD, sputum eosinophilia is associated with response to therapy and has been used for tailored strategies for normalization of sputum eosinophils in order to reduce exacerbation frequency and severity [72]. This may reflect a disruption of the integrity between the interstitial matrix and basement membrane, and inflammation by eosinophils and neutrophils, resulting in the destruction of type VI collagen and a repair response associated with more type VI collagen formation. These biological processes deep within the matrix of the lung, may be associated with COPD phenotypes, disease progression, and events such as exacerbations. In direct alignment, the balance between type VI collagen formation and degradation, was shown to be significantly changed as measured by the same protein biomarkers in serum, in COPD patients with exacerbations [56, 73, 74]. Furthermore, type VI collagen remodeling as measured by these biomarkers has been associated with disease progression, defined by change in FEV_1_, and mortality in COPD patients from the ECLIPSE cohort [65]. The role of type VI collagen in lung pathophysiology still remains to be completely understood and presented at a molecular level, albeit these independent observations suggest that type VI collagen is of particular relevance for lung pathobiology.

### Limitations

This report has several limitations. The data shown was analysed in a fairly limited number of subjects, and the statistical power is further reduced by sub-division of COPD patients into phenotypes, and healthy subjects into smokers and non-smokers. The study did not include a well-defined control group of never-smoking controls with emphysema, which could have further supported the findings related to biomarker elevations of the emphysema phenotype if similar results were found.

A number of observations of elevated biomarkers were not found to be statistically significant between COPD patients and non-COPD smoker control subjects in this analysis. This may in part be explained by the relatively few smoking controls included in the analysis, as visual inspection of the data as shown in the figures indicate a difference which may have the potential to reach statistical significance had the statistical power been higher.

The findings of the present analyses were not validated in a separate validation cohort for the purpose of this report. However, recent published report with the same biomarkers in other well-known COPD cohorts support the findings that C6M and EL-NE are significantly associated with lung function in COPD [73, 74], and have been found to be elevated during exacerbations in a smaller study [56].

Construct validity of blood-based biomarkers is often questioned, as definitive evidence that a particular biomarkers indeed does originate from a certain organ or disease mechanism is often scarce. As the main clinical phenotype under study in the COPDGene cohort is lung disease, it appears reasonable to assume that the origin is the lungs, although elastin and collagen are both abundant in other major organs of the body such as the skin. The role of elastin in the alveoli is described in the literature, and results indicate that particularly elastin is a major target in the pathogenesis of emphysema [75], which supports the finding of emphysema associated with EL-NE.

Previous research to link these protease-specific ECM-fragments with the lungs have resulted in a number of reports which support the hypothesized association. The biomarkers analysed in this report have, in addition to in COPD, been found to be significantly elevated in other respiratory diseases, such as idiopathic pulmonary fibrosis [50, 76, 77] and lung cancer [50], indicating that these fragments are likely to originate from pathological turnover of lung tissue, yet no definitive proof currently exists.

## Conclusion

These data suggest that type VI collagen turnover and elastin degradation by neutrophil elastase are associated with COPD-related inflammation and emphysema. Serological assessment of type VI collagen and elastin turnover may assist in identification of selected phenotypes likely to be associated with more progression and more amenable to precision medicine for clinical trials.

## Abbreviations

6MWD: 6-min walk distance
AAT: Alpha-1 antitrypsin
BMI: Body mass index
C1M: Degradation fragment of collagen type I generated by MMPs
C3M: Degradation fragment of collagen type III generated by MMPs
C6M: Degradation fragment of collagen type VI generated by MMPs
COPD: Chronic obstructive pulmonary disease
CT: Computed tomography
ECLIPSE: Evaluation of COPD longitudinally to identify predictive surrogate end-points
ECM: Extracellular matrix
ELISA: Enzyme-linked immunosorbent assays
EL-NE: Degradation fragment of elastin generated by neutrophil elastase
FEV_1_: Forced expiratory volume in 1 s
FVC: Forced vital capacity
GOLD: Global initiative for chronic obstructive lung disease
IQR: Inter-quartile range
LAA: Low attenuation area
MCS: Mental component score, SF-36
MMP: Matrix metalloproteinase
PCS: Physical component score, SF-36
Pro-C6: C5 domain of collagen type VI released during formation
SD: Standard deviation
